# Comparison of Three Extraction Techniques for the Determination of Volatile Flavor Components in Broccoli

**DOI:** 10.3390/foods9040398

**Published:** 2020-03-31

**Authors:** Martyna Natalia Wieczorek, Małgorzata Majcher, Henryk Jeleń

**Affiliations:** Faculty of Food Science and Nutrition, Poznań University of Life Sciences, 60-624 Poznań, Poland; martyna.wieczorek@up.poznan.pl (M.N.W.); majcherm@up.poznan.pl (M.M.)

**Keywords:** broccoli, flavor, aroma, GC-O, GC×GC-ToFMS

## Abstract

To analyze aroma active components in a food product, the crucial step is to select a suitable extraction technique. It should provide isolation of all components responsible for aroma creation, without the formation of any artifacts during the procedure. Preferably, the extraction method should yield analyzed compounds in detectable levels. The presented study aimed to compare three popular extraction techniques used in flavor studies: solid-phase microextraction (SPME), solvent-assisted flavor evaporation (SAFE), and simultaneous distillation extraction (SDE) in order to isolate aroma components from broccoli (*Brassica oleracea L*. var. *italica*). Obtained extracts were analyzed by gas chromatography-olfactmetry (GC-O) to determine compounds with aroma activity as well as gas chromatography-mass spectrometry (GC-MS) and comprehensive two-dimensional gas chromatography time-of-flight mass spectrometry (GC×GC-ToFMS) to identify them. Thirty-four aroma active compounds were detected in broccoli by the applied techniques. SPME and SAFE together gave the full profile of aroma active components on chromatograms from GC-O, without artifacts that occurred in the SDE extract. SPME was particularly useful in the identification of early eluting compounds, while SAFE enabled isolating compounds with relatively low partition coefficients. Despite all the disadvantages of the SDE method, it leads to the identification of pyrazines, which were important contributors to the overall aroma.

## 1. Introduction

The crucial step in the determination of aroma active components in food products is the selection of suitable extraction techniques. In the case of broccoli, there are a limited number of works focusing on the aroma-active compounds. In order to analyze the key odorants responsible for the aroma of broccoli, it is important to select the most suitable technique that allows isolating all compounds contributing to the characteristic aroma, without artifacts, and yet reflect the “real” aroma profile of broccoli. Preferably, it should be an exhaustive method, as partition-based methods can favor selected compounds only.

Different techniques were applied in order to extract volatiles’ components from *Brassica* vegetables: collecting volatiles in high temperature [1,2,3], simultaneous distillation extraction (SDE) [4,5], sorbent (Tenax) trapping [6], solvent extraction [7] and also solid-phase microextraction (SPME) [8,9]. However, no studies were found that would use solvent-assisted flavor evaporation (SAFE) to isolate volatiles from *Brassica* vegetables. SAFE extraction allows for effective volatile isolation at low temperatures, thus avoiding potential flavor modification or artifacts formation [10]. Traditionally, the widely used SDE technique is versatile and relatively simple, however the high temperature applied during the process, as well as long extraction time, might lead to artifact formation. Both methods require a solvent application and are relatively time-consuming. However, the extract can be stored at low temperatures for a long time and analyzed for as many times as necessary without quality changes. SPME requires the sample preparation indirectly before gas chromatography (GC) analysis. Thus, it does not make it possible to store the extract. However, it should not be forgotten that the SPME technique has also many important benefits, like simplicity, sensitivity and extremely short extraction time. This technique is commonly applied for the isolation of aroma compounds and also for quality purposes, such as to monitor changes in volatiles during storage, volatiles’ profiling, or metabolomics purposes.

Extracts of volatile compounds are subsequently analyzed by gas chromatography techniques. Gas chromatography-olfactometry (GC-O) to characterize an aroma profile of food products is a technique that is often described as a breakthrough in aroma research, which is caused by the association of the resolution power of capillary GC with the selectivity and sensitivity of the human nose. It is worth mentioning that the human nose is able to detect the odouriferous compound at the amount of 10^−17^ g, while detectors commonly used in gas chromatography required at least 10^−13^ g [11]. Gas chromatography with mass spectrometry is a technique used in order to identify aroma-active substances. In gas chromatography-mass spectrometry (GC-MS) the high separation power of GC is complemented by the identification capabilities of the MS. This technique is extremely useful, however, it has limitations, such as sensitivity and problems with coelution in complex matrices. Two-dimensional gas chromatography (GC×GC) can overcome such limitations and is very useful as an additional technique used in aroma-active compounds’ identification when coupled to high speed detectors such as time-of-flight (ToF) mass spectrometers.

The aim of the presented study was to select the most appropriate technique to isolate volatiles from fresh, uncooked broccoli florets, which would be used for the identification of key aroma compounds in subsequent research. The results presented for broccoli would have broader importance for all vegetables from the *Brassicaceae* family. GC-O was used for the identification of odoriferous compounds, whereas GC-MS and two-dimensional gas chromatography time-of-flight mass spectrometry (GC×GC-ToFMS) were used for the identification of odorants and volatile compounds.

## 2. Materials and Methods 

### 2.1. Samples and Reagents

The broccoli was bought fresh from a local grocery shop and kept in a refrigerator at 6 °C until analysis (one day). Dichloromethane, methanol, SPME fiber divinylbenzene/ carboxen/ polydimethylsiloxane (DVB/CAR/PDMS) and all used analytical standards were purchased from Sigma Aldrich (Poznań, Poland). 

### 2.2. Isolation of Volatile Components from Broccoli

#### 2.2.1. Solvent-Assisted Flavor Evaporation (SAFE)

Broccoli sample was blended and 50 g of pulp was weighed and transferred to a 500 mL Erlenmeyer flask, to which 200 mL of dichloromethane was added, and the content in the flask was stirred with a magnetic stirrer for 2 h. After this time the solution was filtered and subjected to volatiles extraction in a SAFE apparatus (Glassbläserei Bahr, Manching, Germany) [12]. During the procedure the temperature of the sample was kept at 40 °C and vacuum was provided using a dual stage rotary vane pump (RV-5, Edwards, UK). The sample was poured into addition funnel and the liquid nitrogen was added into the cooling trap. After the sample addition, the extraction lasted for 30 min. Then, the extract was dried over anhydrous sodium sulphate and concentrated to about 1.5 mL in Kuderna-Danish concentrator (Sigma-Aldrich, Poznań, Poland) and immersed in a 40 °C water bath. The extraction was performed according to procedure described by Majcher and Jeleń [10].

#### 2.2.2. Simultaneous Distillation Extraction (SDE)

For SDE extraction, a Clavenger type apparatus was used. A sample of 50 g of blended broccoli was placed in a 500 mL flask and mixed with 70 mL of distillated water. The round-bottom flask was attached to the bottom of SDE apparatus and placed in a heating mantle. A pear-shaped 50 mL flask containing 20 mL dichloromethane as extraction solvent was connected to the second arm of the instrument. The distillation/extraction was performed for 2 h and after that, the extract was dried over anhydrous sodium sulphate and concentrated to about 1.5 mL in a Kuderna-Danish concentrator immersed in 40 °C water bath. The extraction was performed according to procedure described by Majcher and Jeleń [10].

#### 2.2.3. Solid-Phase Microextraction (SPME)

The SPME fiber (DVB/CAR/PDMS) was preconditioned in the injection port in 270 °C per 1 h before analysis. 4 g of blended broccoli sample was placed in a 20 mL vial. The extraction was performed at 50 °C, the sample was preheated for 5 min and then the fiber was exposed in the vial for 10 min. The extraction was performed according to Wieczorek and Jeleń methodology [13]. All analyses were run in triplicate.

### 2.3. Chromatographic Analyses 

#### 2.3.1. Gas Chromatography–Olfactometry (GC-O) Analysis

Gas chromatography–olfactometry was performed on HP 5890 II (Hewlett-Packard, Waldbronn, Germany) chromatograph using two capillary columns of different polarities: SPB-5 (30 m × 0.32 mm × 0.50 µm, Supelco, Bellefonte, PA, USA) and Supelcowax 10 (30 m × 0.25 mm × 0.50 µm, Supelco Bellefonte, PA, USA). The operating conditions were as follows: initial oven temperature 40 °C (1 min), then 6 °C/min to 200 °C and 25 °C/min to 280 °C (3 min). For all peaks and flavor notes occurring at a specific retention times, retention indices (RI) were calculated to compare results with those obtained by GC/MS and data in literature. Retention indices were calculated for each compound using homologous series of C5–C24n-alkanes [14].

#### 2.3.2. Gas Chromatography-Mass Spectrometry (GC-MS) Analysis

Compounds’ identification was performed on gas chromatograph coupled with triple quadrupole mass spectrometer (7890A/7000B, Agilent Technologies, Santa Clara, CA) working in a single quadrupole mode. The mass spectrometer was operated in electron ionization (EI) mode at 70 eV, in a scan range of *m/z* 33−333. Samples were injected in a splitless mode onto SPB-5 (30 m × 0.20 mm × 0.50 µm, Supelco Bellefonte, PA, USA) column. The oven temperature program during analysis was as follows: 40 °C (1 min), then 6 °C/min to 200 °C and 25 °C/min to 280 °C (3 min). Temperatures of the injector and mass spectrometer ion source were 250 °C and 230 °C, respectively. Helium was used as a carrier at a flow of 0.8 mL/min. Data acquisition and analyses were performed using the Agilent Technologies MassHunter Workstation (B 07.00, Santa Clara, CA, USA) software. Tentative identification was accomplished using the National Institute of Standards and Technology (NIST, Gaithersburgh, MD, USA) library (version 2.0) of mass spectra.

#### 2.3.3. Comprehensive Two-Dimensional Gas Chromatography Time-of-Flight Mass Spectrometry (GC×GC-ToFMS) Analysis

Comprehensive two-dimensional gas chromatography analysis was performed on a GC×GC–ToF-MS system (Pegasus 4D, LECO, St. Joseph, MI, USA). The GC was equipped with a DB-5 primary column (25 m × 0.2 mm × 0.33 μm, Agilent Technologies, Santa Clara, CA, USA) and Supelcowax 10 (1.2 m × 0.1 mm × 0.1 μm, Supelco, Bellefonte, PA, USA) as a secondary column. The injector temperature was set at 250 °C and injection was performed in splitless mode. The gas flow was set at 0.8 mL/min. The entry oven temperature was programed as follows: 40 °C (1 min) 60 °C/1 min to 200 °C (0 min) 25 °C/1 min to 235 °C (5 min). Secondary oven: 65 °C (1 min) 6 °C/1 min to 225 °C (0 min) 25 °C/1 min to 260 °C (5 min). Transfer line temperature was 260 °C. The modulation time was 4 s. The time-of-flight mass spectrometer was operated at a mass range of *m*/*z* 33–383 and detector voltage −1700 *V* at 150 spectra/s. The data were collected and processed using LECO ChromaTOF v.4.40 Total analysis time was 34.07 min. For SAFE and SDE samples, 3 min of solvent delay was applied.

## 3. Results and Discussion

### 3.1. Evaluation of SAFE, SDE and SPME Extracts by GC-O 

Three different extraction techniques were compared in order to evaluate their suitability for the determination of aroma components in broccoli. Two of them were exhaustive techniques: SAFE, SDE, and one non-exhaustive: SPME. Those were selected based on their popularity in research related to aroma active components in food products. SDE, which is the most traditional extraction method, widely applied for food products was compared with the modern SAFE technique, which does not require a high-temperature application. SPME, on the other hand, is very useful in volatile analysis. It is a solventless technique, especially convenient for analysis of small to medium-mass substances. In comparison with other non-exhaustive methods, it is characterized by its simplicity and also it does not require any specific equipment for its performance. Extracts were subsequently analyzed by different chromatographic methods. First, GC-O was employed to determine aroma active compounds, and GC-MS to identify them. Then GC×GC-ToFMS was employed to identify components not visible on GC-MS chromatogram. Additionally, results from GC×GC-ToFMS analysis were analyzed in order to profile volatiles from the three applied extraction techniques. Potent odorants (31) were identified in the SAFE extract, 27 in the SDE extract and 21 after applying SPME (Table 1). The SAFE technique gave the best results in the detection of aroma compounds, compared to the other two methods. SPME was important for identifying compounds eluting in the first few minutes, which coelute with a solvent peak on a GC-MS chromatogram and in the other extractions that are not visible due to solvent delay in the mass spectrometer. The first 3 min brought some characteristic odors in all extracts: “sulfur” resulting from the presence of methanethiol—a compound with extremely low odor threshold (0.2 µg/L) [15]. Putrid, cabbage-like odor notes were perceived in SDE and SPME extracts caused by dimethyl sulfide (odour threshold 12 ppb [16]) and “buttery” odor present in all samples, assigned as 2,3-butanedione. Those first eluting compounds were particularly relevant for the flavor, because of their low odor threshold and characteristic aromas. Identification of early eluting compounds was possible only by applying the SPME solventless technique.

All sulfides: dimethyl sulfide, dimethyl disulfide, dimethyl trisulfide, and dimethyl tetrasulfide gave more intense aroma in the case of the SDE extract, than in the two remaining solutions. This was probably due to the high-temperature applied during extraction, leading to the degradation of their precursors and/or thermal formation of di-, tri-, and tetrasulfides. Moreover, the concentration of sulfides was lower in SAFE compared to SDE and SPME, illustrating the influence of sample temperature on the formation of these compounds. In the SAFE extract, S-methylmethanethiosulphonate was presented in higher amounts (relatively high peak area) than in the two other extractions. High amounts of this component might be caused by low temperature extraction and fast enzymes inactivation, caused by adding the solvent right after tissue disruption. In SDE, the broccoli pulp was mixed with water in the first step, which created the possibility of enzymatic reactions and, moreover, the high temperature may lead to the thermal-induced sulfides formation. In SPME, the enzymes were probably active during the extraction procedure however, the odor intensity was lower than in SDE. 

Another meaningful difference was the “popcorn” aroma in the SDE extract, apparently caused by high temperature application, which suggests the possibility of artifact formation during this procedure. All other differences were mostly caused by the fact, that SAFE and SDE techniques are exhaustive extraction types, while SPME allows for the extraction of the compounds from the volatile phase depending on their partition coefficients. Additionally, it is a selective technique, the selectivity of which is fiber-dependent, so the volatile profile could differ from the two remaining isolation techniques. 

Summarizing, the best option is the application of both SAFE aided with SPME extraction to achieve a reliable odor active compounds profile. Although SDE was popular and often used in early studies with *Brassica* vegetables, the high temperature of extraction and long extraction time makes it unsuitable in the analysis of fresh vegetable volatiles. However, in certain cases, SDE can provide bigger peaks of thermally stable compounds than the other two techniques and can aid in their identification.

Some of the identified aroma components in broccoli have already been described in literature. Methanethiol is a meaningful aroma component in cheese, as well as different alcoholic beverages [15] and also appeared to be the primary contributor to the objectionable odor that broccoli develops when held in anaerobic conditions [17]. The odour of this compound was described as “intensely putrid, fecal-like aroma” and might be the reason for the rejection of broccoli by many consumers. Dimethyl disulfide has been detected as a major volatile sulfur compound in the headspace of broccoli [16] as well as cabbage [18], asparagus, corn, and tomato paste [19] and is considered to be a secondary product of primary C-S lyases action on S-methyl-L-cysteine sulfoxide [20]. Dimethyl trisulfide was also detected in *Brassica* vegetables before, such as cabbage or broccoli [16]. Methyl thiocyanate, was also previously identified in broccoli stored under a low oxygen atmosphere [16] and in cabbage [4]. Hexyl isothiocyanate was the only identified isothiocyanates in the extracts and was the source of nice aroma “broccoli-like”; this component was previously detected in Japanese and Kenyan radish [21].

### 3.2. Evaluation of SAFE, SDE and SPME Extracts by GC×GC 

All extracts were analyzed by GC×GC-ToFMS (Figure S1 in supplemental materials) and the results are shown in Table 2. The analysis was performed in order to compare volatile profiles among different samples. Those results were also useful in identification of aroma-active components in Table 1. The use of SDE, SAFE and SPME allowed for the identification of 120 volatile compounds extracted from fresh broccoli. All of them were identified tentatively by comparison with NIST spectra after deconvolution using Chromatof 4.40 software. Compounds with the probability of > 700 were listed in the table along with their molecular masses.

**Figure 1:**
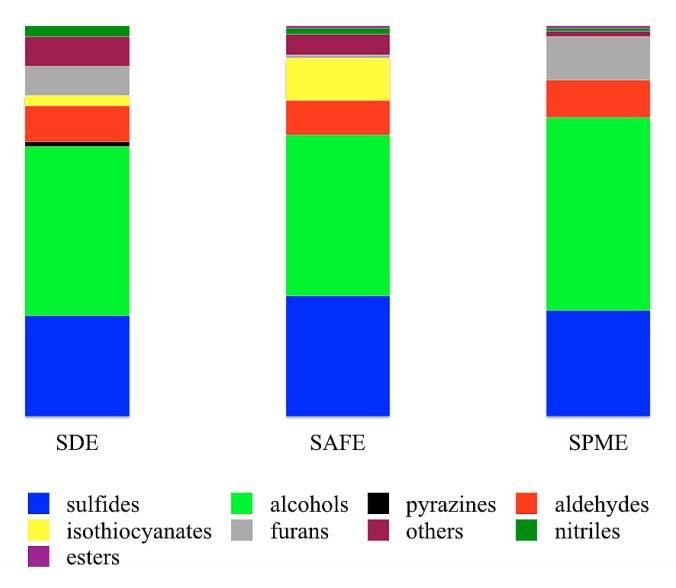
Main groups of volatile compounds in raw broccoli obtained using different extraction techniques. Graphs created based on area % of peaks identified using two-dimensional gas chromatography time-of-flight mass spectrometry (GC×GC-ToFMS).

All extraction techniques gave a majority of alcohols, which consist of at least 40% of all chemical groups extracted (Figure 1). The second most abundant fraction was sulfur components (mainly sulfides). The meaningful differences between extraction techniques were observed in pyrazines and furans concentration. Pyrazines are present almost exclusively in the SDE extract; however, it is worth highlighting that the aroma of pyrazines (green peas, etc.), observed in GC-O analysis is present in all samples (see Table 1). On chromatograms, the pyrazines are visible only in SDE extracts, which clearly indicates that despite all the disadvantages of this technique it might be helpful for the identification of some compounds not visible in milder techniques (SAFE, SPME). There is also a high concentration of furans in SPME and SDE extracts. 

SPME is specifically important for the determination of early eluting compounds, like methanethiol, dimethyl sulfide etc., which coelute with the solvent and, therefore, is not visible with solvent delay in splitless injections. Sulforaphan a bioactive molecule with proven positive health benefits that was present only in the SAFE extract, probably caused by the non-stable nature of this compound. SDE applied high temperature, which might lead to its destruction, while in SPME it might be extraction temperature, extraction time, or most likely the low value of distribution constant that influences the migration of compounds to headspace. It is worth noting the proportion between sulfides in particular extracts. The percentage of dimethyl disulfide is definitely higher in SPME than in the two other methods. Dimethyl trisulfide is on a similar level in all analytes, however the percentage is slightly higher in SPME (Table 2). Dimethyl tetrasulfide is present in very low concentrations in SPME compared with SDE and SAFE. Dimethyl pentasulfide is not present in SPME extracts, while it is relatively high at 3.6% in the SDE extract and at 0.4% in the SAFE extract. The absence of high molecular sulfides in the SPME extract might be caused by their low Henry’s constant and molecular weight, as SPME is more suitable for low molecular weight compounds isolation. 

Analysis of volatiles in plants is a complex issue. The composition of volatile metabolites is unstable and dependent on many factors, such as storage conditions, cultivars or technological processes [16]. This makes it extremely important to choose an appropriate technique to isolate those components. Ideally, this technique should not modify the volatile composition by high temperature, long extraction times, etc. Moreover, in the case of aroma-active components determination, those factors are especially important. In the presented study, among the three applied techniques, the most suitable for the determination of aroma-active components was the SAFE method, mostly because it is an exhaustive extraction technique and does not require high temperatures as with the SDE technique. Most importantly, SAFE extraction led to the isolation of the greatest number of aroma active components. However, in the case of non-targeted analysis of volatiles in *Brassica*, all applied techniques might be useful, because the composition of extracted components differs between them. The common feature of all extracts was the majority of alcohols and sulfides. The main differences were related to isothiocyanates and pyrazines. Isothiocyanates were most effectively isolated by the SAFE method, while pyrazines by the SDE technique. The choice of appropriate extraction technique is of the highest importance in terms of flavor compounds composition and representativeness of the sample aroma. However separation of compounds using the right column for compounds of different character, polarity and belonging to different chemical classes is also important. Analysis of aroma compounds in flavor research is carried out often on two columns of different separation mechanisms to unequivocally resolve and identify the compounds of interest. GC×GC provides spatial separation of compounds required in this type of research. 

The only previous attempt of analysis of aroma-active compounds in broccoli was performed by Ulrich et al. [8]. Two extraction techniques were compared—SPME and the dynamic headspace sampling on a Tenax trap. Twelve aroma-active compounds were noted, however most of them were not identified. The comparison of SAFE, SDE and SPME suitability for GC-O analysis was performed by Majcher and Jeleń [10] in order to determine components responsible for the aroma of extruded potato snacks and the results led to a similar conclusion as the present study. 

## 4. Conclusions

The research presented is the first step towards analyzing key aroma compounds in broccoli and other *Brassica* vegetables. It showed the advantages and limitations of three important extraction techniques when they were applied to isolate volatiles from vegetables. To the best of our knowledge, it is the first attempt at broccoli volatilome analysis by the SAFE technique. There have been publications concerning SPME or SDE applications. However, one must remember that SPME is a partition-based extraction technique in which the profile of extracted compounds is highly dependent on fiber coating used. As new fiber coatings are developed, some of them dedicated to high-mass compounds or in vivo extraction, it might be possible to obtain more information on analyzed compounds due to their selectivity. For flavor studies conducted with GC-O, SPME cannot be the only method of aroma investigation, although it is the favorite for odorant target analyses due to pre-concentration capabilities. 

Three different extraction techniques were compared to evaluate their suitability for the isolation of aroma components in raw broccoli. SAFE seemed to be the most useful technique, due to the low extraction temperature and ability to extract the most compounds. SDE allowed for the isolation of numerous aroma-active components, however, the high temperature applied during the process induced artifacts formation. SPME extraction allows identification of low boiling compounds co-eluting with solvents used in other methods. The main benefit besides simplicity, rapidity and low cost is its “green” character. This technique does not require any toxic solvents, which is very especially relevant nowadays. Traditional key aroma determination strategies used SAFE as a basic method. This is mostly because the exhaustive extraction technique provides extracts for the flavor dilution factor (FD) determination. However, SAFE suffers from the presence of a solvent peak and problems with the analysis of low-boiling temperature compounds. For SPME analyses there is no possibility to dilute SPME extract, though some approaches use split regulation for this purpose. 

This study showed the complexity of broccoli matrix flavor and advantages and disadvantages of some of the most commonly used aroma extraction techniques. Based on the presented experiments, SAFE is a basic technique and SPME a supplementary method for these types of sample.

## Figures and Tables

**Table 1:** Compounds identified using gas chromatography-olfactmetry (GC-O) and gas chromatography-mass spectrometry (GC-MS) in raw broccoli after extraction using three methods: solvent-assisted flavor evaporation (SAFE), simultaneous distillation/extraction SDE) and solid-phase microextraction (SPME). *—identity confirmed with authentic standard analysis.

Odour Type	Name	SAFE	SDE	SPME	RI DB5	Major Fragment Ions *(m/z/)*
sulfur, burnt	methanethiol *	+	+	+	>500	47 (100) **48(89)**; 45(47); 46(12); 15(10)
putrid, cabbage	dimethyl sulfide *	-	+	+	>500	**62(100**); 47(95); 45(41); 46(36); 61(33)
buttery	2,3-butanedione *	+	+	+	>600	43(100); **86(18)**; 46(88); 15(44); 44(28)
rancid, sulfur	2/3- methyl butanal	+	+	-	655	41(100); 29(93); 57(88); 58(58); 27(42);
garlic	methyl thiocyanate *	+	+	+	665	**73(100)**; 72(58); 45(36); 44(32); 46(32)
buttery	2,3-pentanedione *	+	-	-	707	43(100); 29(61); 57(33); 27(26); **100(11)**
garlic	dimethyl disulfide *	-	+	+	720	**94(100)**; 79(57); 45(48); 46(25); 47(19)
cabbage	unknown	+	-	-	790	-
grass	hexanal *	+	+	+	795	44(100); 56(81); 41(69); 43(55); 57(38)
garlic	unknown	+	+	+	804	-
cabbage, sweaty	2/3-methyl butanoic acid	+	-	+	847	74(100); 57(64); 29(62); 41(53); 27(32)
grassy, green	3-hexen-1-ol *	+	+	+	848	41(100); 67(59); 39(39); 55(34); 69(27)
rancid	Z-(4)-heptenal	+	+	+	898	44(100); 70(94); 43(84); 41(67); 55(59)
boiled potatoes	methional *	+	+	+	905	44(100); **104(51)**; 47(43); 76(33); 45(28)
popcorn	2-acetyl-1-pyrroline	-	+	-	920	43(100); 41(54); 42(24); 83(13); 39(11)
cabbage	unknown	+	-	-	951	-
putrid, cabbage	dimethyl trisulfide *	+	+	+	975	**126(100)**; 45(59); 79(51); 47(36); 64(22)
geranium	1,5-octadien-3-one	+	+	+	986	-
cabbage	S-methylmethanethiosulphinate	+	-	-	995	64(100); 47(74); **110(61)**; 45(46) 32(39)
broccoli	unknown	+	+	-	1032	-
honey	phenylacetaldehyde *	+	+	-	1046	91(100); 92(29); **120(28)**; 68(18); 39(74)
earthy, roasted	2-ethyl-3,5-dimethylpyrazine	+	+	+	1054	135(100); **136(78)**; 42(36); 39(28); 54(20)
sauerkraut	S-methyl methanethisulphonate *	+	-	-	1071	47(100); 81(87); 63(73); 79(68); **126(66)**
green peas	2-isopropyl-3-methoxy pyrazine *	+	+	+	1094	137(100); **152(42)**; 124(26); 138(12); 109(11)
broth like	unknown	+	-	-	1119	-
hop	unknown	+	+	+	1133	-
roasted	unknown	+	+	-	1148	-
cucumber	(E)-2-nonenal *	+	+	+	1159	43(100); 41(99); 29(76); 55(76); 70(72)
earthy, roatsted	2-s-butyl-3-methoxypyrazine	+	+	+	1178	138(100); 124(65); 151(45) 137(31); 123(17)
pepper like	2-isobutyl-3-methoxy pyrazine	+	+	+	1185	108(100); 135(14); 107(10); 67(9); 41(9)
broccoli	hexyl isothiocyanate *	+	+	+	1209	43(100); 12(61); 41(46); 72(27); 29(24)
broth like	unknown	-	+	+	1217	-
soup	(E)-2-decenal *	+	+	-	1236	43(100); 41(88); 55(70); 70(70); 29(67)
fatty	(E, E) -2,4-decadienal *	+	+	-	1323	81(100); 41(51); 39(22); 27(22); 29(21)

RI SPB-5 Retention indices calculated for DB-5 phase type column; in major fragments ions column base peak was provided (with intensity 100(%)), as well as molecular ion (if observed, bolded) and other major fragments.

**Table 2:** Volatile compounds identified by GC×GC-ToFMS (tentative identification using NIST mass spectra library) from SAFE. SDE and SPME extracts. nd: not determined. Quantities expressed as percentage value using two decimal points. Compounds with peak area % > 0.01% shown. Compounds listed according to their increasing molecular weight (MW).

Compound	Molecular Weight	SDE %	SAFE %	SPME %
methanethiol	48	S	s	0.26
ethanenitrile	55	S	s	0.26
dimethyl sulfide	62	S	s	2.42
1,3-pentadiene, (E)-	68	0.76	0.03	nd
1,3-pentadiene, (Z)-	68	0.80	nd	nd
1,4-pentadiene	68	0.13	nd	0.03
butanenitrile	69	0.61	nd	nd
thiocyanic acid, methyl ester	73	2.30	1.12	4.38
1-butanol	74	0.32	0.04	nd
methyl ethyl sulfide	76	nd	nd	0.02
dimethyl sulfoxide	78	2.93	5.38	0.04
pyrazine	80	0.28	nd	nd
2-pentenenitrile	81	0.18	nd	nd
methallyl cyanide	81	nd	0.05	nd
furan, 2-methyl	82	s	s	0.06
butanenitrile, 3-methyl-	83	0.47	nd	nd
1-penten-3-one	84	0.55	0.85	0.73
2-pentenal, (E)-	84	0.67	1.20	0.26
1-penten-3-ol	86	4.84	9.31	10.01
2-penten-1-ol. (E)-	86	11.86	14.34	10.03
butanal, 2-methyl-	86	nd	0.51	nd
pentanal	86	0.38	1.52	nd
sulfide, allyl methyl	88	0.02	nd	nd
dimethyl sulfone	94	0.05	1.62	0.51
disulfide, dimethyl	94	5.50	3.19	13.00
pyrazine. methyl-	94	0.40	0.01	nd
2,4-hexadienal, (E,E)-	96	0.60	0.28	0.19
furan, 2-ethyl-	96	1.84	0.65	10.29
furfural	96	3.76	nd	nd
hexanenitrile	97	0.09	0.06	0.19
pentanenitrile, 4-methyl-	97	s	s	0.01
2-hexenal, (E)-	98	1.39	1.06	3.63
3-hexanal	98	nd	0.55	0.57
thiophene, 2-methyl-	98	0.05	0.04	0.03
thiophene, 3-methyl-	98	0.02	nd	nd
2-hexen-1-ol, (E)-	100	8.51	6.02	0.02
3-hexen-1-ol, (Z)-	100	5.62	3.51	23.58
hexanal	100	0.52	0.31	3.10
isopropyl isothiocyanate	101	0.03	nd	nd
1-hexanol	102	9.70	6.48	4.91
S-methyl propanethioate	104	0.03	nd	nd
benzaldehyde	106	0.51	1.09	0.46
pyrazine, ethenyl-	106	0.02	nd	nd
hexanedinitrile	108	0.02	0.01	nd
pyrazine, 2.3-dimethyl-	108	0.10	nd	nd
pyrazine, 2.5-dimethyl-	108	0.20	nd	nd
2,4-heptadienal, (E,E)-	110	2.05	1.21	0.26
furan, 2-ethyl-5-methyl-	110	0.10	nd	0.01
furan, 2-propyl-	110	0.32	nd	0.02
hexanenitrile, 5-methyl-	111	0.10	0.03	0.28
2(5H)-furanone, 5-ethyl-	112	0.71	1.65	0.14
3-hepten-2-one	112	0.52	0.04	nd
thiophene, 2-ethyl-	112	0.46	0.38	2.87
1-butene, 4-isothiocyanato-	113	1.75	9.83	0.01
heptanal	114	0.45	0.08	0.15
octane	114	0.26	nd	nd
butane, 1-isothiocyanato-	115	nd	nd	0.01
butane, 2-isothiocyanato-	115	0.10	nd	nd
butanenitrile, 4-(methylthio)-	115	0.06	nd	nd
isobutyl isothiocyanate	115	nd	nd	0.01
benzyl nitrile	117	0.13	0.21	0.05
indole	117	1.67	0.03	nd
butanethioic acid, S-methyl ester	118	0.04	nd	nd
benzeneacetaldehyde	120	0.69	0.64	0.72
pyrazine, (1-methylethenyl)-	120	0.01	nd	nd
phenylethyl alcohol	122	nd	0.66	0.17
pyrazine, trimethyl-	122	0.05	nd	nd
1,2,4-trithiolane	124	0.11	1.33	0.06
2,4-octadienal, (E,E)-	124	0.66	nd	nd
cyclohexane, isocyanato-	125	0	0.01	nd
butane, 1-isothiocyanato-3-methyl-	126	0	0.01	0.01
dimethyl trisulfide	126	2.33	2.57	3.37
S-methyl methanethiosulphonate	126	2.80	9.53	0.08
cyclopentyl isothiocyanate	127	0.01	0.06	nd
hexane, 1-isocyanato-	127	nd	0.03	0.01
3-hexen-1-ol, formate. (Z)-	128	2.13	0.77	0.82
butanoic acid, 2-propenyl ester	128	nd	0.58	nd
heptane, 2.4-dimethyl-	128	nd	0.28	nd
octanal	128	0.10	nd	nd
2-methylbutyl isothiocyanate	129	0.14	nd	nd
pentanenitrile, 5-(methylthio)-	129	nd	0.51	nd
n-pentyl isothiocyanate	129	0.05	0.02	0.01
hexanoic acid, methyl ester	130	nd	nd	0.49
1H-indole, 3-methyl-	131	0.43	0.01	0.01
benzenepropanenitrile	131	0.89	0.34	0.08
S-methyl pentanethioate	132	0.01	0.03	nd
benzyl isocyanate	133	nd	0.01	nd
benzothiazole	135	0.05	0.75	nd
3-ethyl-1,5-octadiene	138	0.63	0.17	0.18
benzene, 1,2-dimethoxy-	138	nd	0.51	0.21
furan, 2-pentyl-	138	1.32	0.09	0.73
thiophene, 3.4-diethyl-	140	0.03	nd	nd
cyclohexane, isothiocyanato-	141	nd	nd	nd
2-hexen-1-ol, acetate, (Z)-	142	0.47	nd	nd
nonanal	142	0.23	0.29	0.10
4-methylpentyl isothiocyanate	143	nd	0.06	nd
hexane, 1-isothiocyanato-	143	0.09	0.04	0.01
benzenebutanenitrile	145	0.01	nd	nd
sulforaphane nitrile	145	nd	0.22	nd
1H-indole, 1-methoxy-	147	0.50	nd	nd
benzene, (isothiocyanatomethyl)-	149	0.11	0.41	0.01
methyl pentyl disulfide	150	0.23	0.01	nd
2,4-decadienal, (E,E)-	152	0.67	0.07	0.01
furan, 2-hexyl-	152	0.03	nd	nd
pyrazine, 2-methoxy-3-(1-methylethyl)-	152	0.01	nd	nd
1H-indole-3-acetonitrile	156	0.28	1.15	nd
decanal	156	0.18	0.07	0.01
tetrasulfide, dimethyl	158	4.98	3.71	0.06
methyl n-octyl sulfide	160	0.08	0.01	nd
benzene, (2-isothiocyanatoethyl)-	163	0.52	0.43	nd
methyl n-hexyl disulfide	164	0.03	nd	nd
sulforaphane	177	nd	0.03	nd
sulfide, methyl 1-methyl-2-butenyl	178	0.01	nd	nd
butanoic acid, 3-hexenyl ester. (E)-	184	nd	nd	0.07
pentasulfide, dimethyl	190	3.61	0.41	nd
tetradecane	198	0.13	0.28	nd
pentadecane	212	0.06	0.04	nd
heptadecane	240	0.13	0.22	nd
hexadecanal	240	0.06	0.03	nd
heptacosane	380	0.16	nd	nd
sulfurous acid, octadecyl pentyl ester	404	0.16	0.81	nd

s—solvent delay; nd—not detected.

## Supplementary Materials

The following are available online at https://www.mdpi.com/2304-8158/9/4/398/s1: Figure S1: GC×GC-ToFMS chromatograms of fresh broccoli volatiles acquired using (**A**) SAFE; (**B**) SDE and (**C**) SPME extraction techniques.
