# Supplementary material for: Comparison of Three Extraction Techniques for the Determination of Volatile Flavor Components in Broccoli
Source: Foods. 2020 Mar 31;9(4):398. doi: 10.3390/foods9040398 (PMC7230502; doi:10.3390/foods9040398)
Supplement: Supplementary file 1 [file foods-09-00398-s001.pdf]

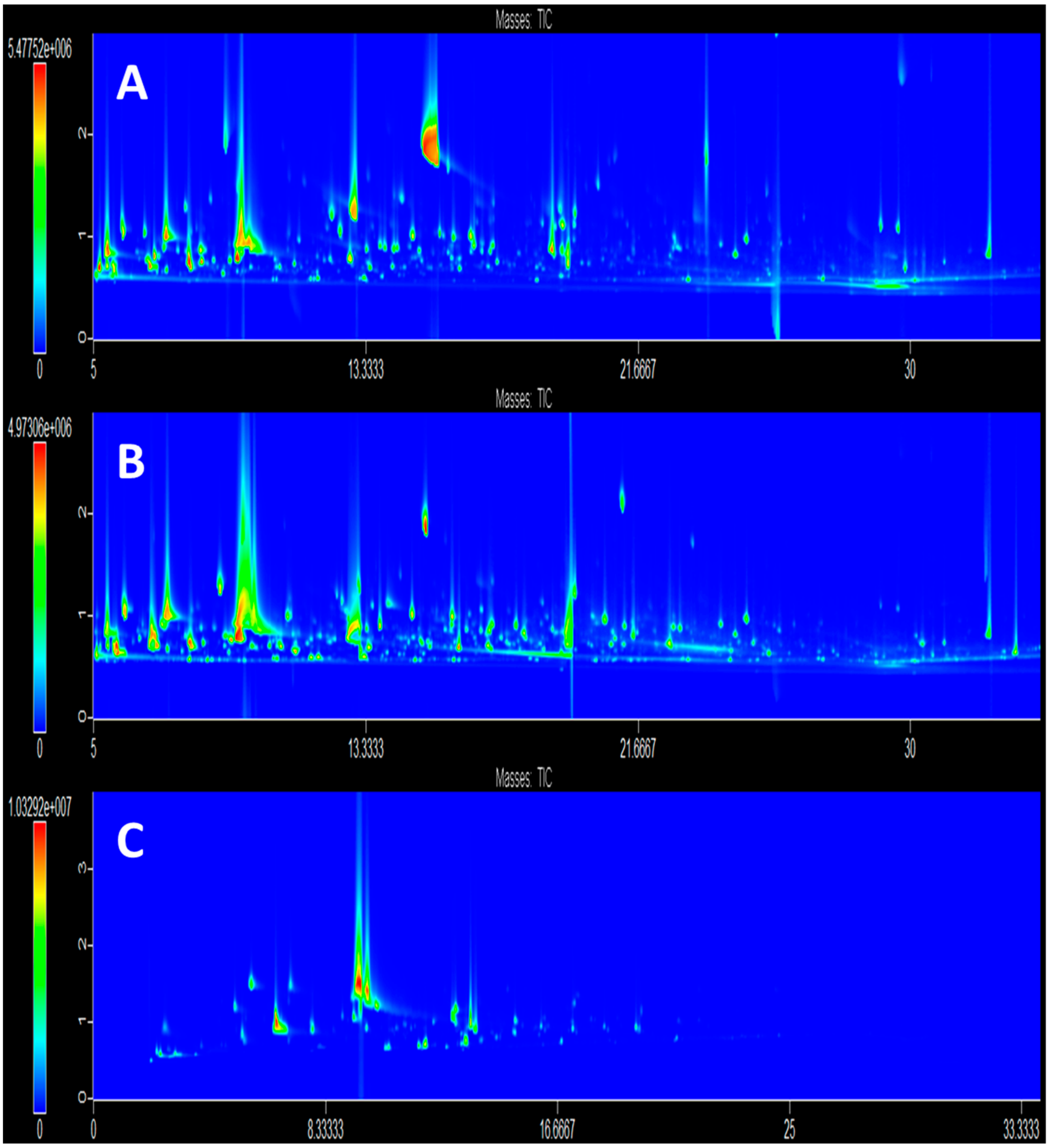

Figure 1. S. GCxGC-ToFMS chromatograms of fresh broccoli volatiles acquired using (**A**) – SAFE; (**B**) – SDE and (**C**) – SPME extraction techniques.
